# Evaluation of conditioned medium from Sertoli cells as a potential inducer of *in vitro* germ cell differentiation of bovine fetal mesenchymal stem cells (MSCs)

**DOI:** 10.1590/1984-3143-AR2024-0094

**Published:** 2025-10-17

**Authors:** Barbara Leiva, Jahaira Cortez, Moises Segunda, Cristian Torres, Alejandro Escobar, Carlos Diaz, Oscar Peralta

**Affiliations:** 1 Facultad de Ciencias Veterinarias y Pecuarias, Universidad de Chile, Santiago, Chile; 2 Escuela de Medicina Veterinaria, Facultad de Agronomía e Ingeniería Forestal, Facultad de Ciencias Biológicas, Facultad de Medicina, Pontificia Universidad Católica de Chile, Santiago, Chile; 3 Faculdade de Medicina Veterinária, Universidade José Eduardo dos Santos, Huambo, Angola; 4 Instituto de Investigación en Ciencias Odontológicas, Facultad de Odontología, Universidad de Chile, Santiago, Chile; 5 Programa de Doctorado en Ciencias, Escuela Internacional de Doctorado, Universidad Nacional de Educación a Distancia – UNED, Madrid, Spain

**Keywords:** mesenchymal stem cells, germ cells, Sertoli cells, Sertoli cell conditioned medium

## Abstract

Due to their self-renewal and differentiation potentials, mesenchymal stem cells (MSCs) may be induced into germ cells (GC) differentiation under *in vitro* conditions. In veterinary medicine, this technology could provide an alternative method to artificial insemination, as well as potentially useful for the conservation of endangered species. Previous studies have reported the use of SCs and MSCs co-culture systems, as well as SCs conditioned medium (SCCM) to induce GC differentiation of human and murine embryonic stem cells (ESCs) and induced pluripotent stem cells (iPSCs). The objective of this study was to evaluate the effect of SCCM as an inducer of *in vitro* GC differentiation of MSCs derived from fetal bovine adipose tissue (AT-MSCs). SCCM was collected from bovine SC cultures generated from adult bull testis. The effect of SCCM on MSCs was analyzed using quantitative PCR (Q-PCR) and flow cytometry. CD73 mRNA levels were decreased (P<0.05) in AT-MSC/SCCM at day 14 of culture compared to control. CD90 and CD105 gene expression were detected during the 21 days of culture; however, relative expression levels were not different (P>0.05) between treated and controls cells. DAZL gene expression was detected on day 21 of culture, as well as a proportion of AT-MSC positive for DAZL at day 21 of culture. OCT4, PIWIL2 and DAZL gene expressions were detected from day 0, 7 and 21 of culture, respectively, as well as a proportion of cells positive for each marker were detected at day 21 of culture. However, similar gene and protein expression levels (P>0,05) were detected between AT-MSCs/SCCM and control cultures. DMC1 gene expression levels were detected from day 7 of culture, and expression levels were not different (P>0,05) between treatment and control cells. Expression patterns of MSC, pluripotent, GC and meiotic markers indicate that SCCM did not induce GC differentiation of AT-MSCs.

## Introduction

Mesenchymal stem cells (MSCs) are defined as adult multipotent cells, morphologically similar to fibroblasts, that display potentials for self-renewal and differentiation (Williams and Hare, 2011). MSCs can be obtained from various tissues, including bone marrow (BM-MSCs), adipose tissue (AT-MSCs), and umbilical cord (UC-MSCs) (Latifpour et al., 2014). These cells possess therapeutic potential mainly based on their immunomodulatory, angiogenic and antibacterial capabilities. Moreover, their capacity to differentiate to functional cells, including germ cells (GCs), has recently been proposed as an attractive strategy for the treatment of human infertility (Nayernia et al., 2006; Wang et al., 2020a). In the case of animal reproduction, derivation of GCs from MSCs could have important applications in livestock reproduction, offering an alternative method to artificial insemination for promotion of genetic dissemination from elite animals (Herrid and McFarlane, 2013). Additionally, this technology could be useful for the conservation of endangered species, allowing for an increase in the size of genetic pools in threatened animal populations (Liu et al., 2013).

The development of appropriate culture conditions for *in vitro* gamete production is essential, as they require a specific microenvironment containing different metabolic factors, molecular pathway factors, sex steroids, nutrition, among others (Zhang et al., 2020). These culture conditions need to recreate the testicular niche in which GCs are found *in vivo*, and potentially to induce transdifferentiation of MSCs into GCs (Huang et al., 2010; Ghaem et al., 2018). Particularly in the testicular niche, Sertoli cells (SCs) play a fundamental role in GCs development by providing nutrient and secreting factors required for their proliferation and differentiation (Huleihel et al., 2007). For example, bone morphogenetic protein-4 (BMP4), whose receptors are predominantly expressed in spermatogonias, exert a differentiating effect on these cells by stimulating the expression of tyrosine kinase receptor (C-KIT) (Rossi and Dolci, 2013; Yang et al., 2016). C-KIT binds to its ligand, stem cell factor (SCF), which is produced by SCs and functions to stimulate the differentiation of spermatogonias into spermatocytes (Unni et al., 2009). Another important factor secreted by SCs is TGFβ3, which function is implicated in the differentiation of GCs (Lui et al., 2003; Skinner and Griswold, 2005). Additionally, the effect of retinoic acid (RA) produced by SCs, favors spermatogonial differentiation through a direct action and an indirect effect mediated by BMP4 secreted by SCs (Hogarth and Griswold, 2010). In addition, RA induces meiotic initiation through controlling the RAR-dependent expression of STRA8 in premeiotic spermatocytes (Rossi and Dolci, 2013; Teletin et al., 2019).

Recently it has been reported that co-cultures of AT-MSCs or BM-MSCs with SCs result in their differentiation of MSCs, reaching early stages of GC development (Segunda et al., 2019, 2023, 2024). Cell co-culture systems can largely simulate the *in vivo* environment, and they facilitate an assessment of the interaction between different types of cells and their environment. These cultures systems also permit the exploration of the mechanisms of action of effector cells over target cells and their potential targets. However, cell co-cultures systems have the disadvantage that cells are mixed in culture, which makes it difficult to study specific cell populations. This drawback may be improved by using conditions media (CM) from effector cells, which contain the specific molecules that mediate their effect, and allow the homogeneity of target cells to be maintained. Therefore, the purpose of this study was to evaluate the effect of CM from SCs (SCCM) as an inducer for *in vitro* differentiation of fetal bovine AT-MSCs into GCs.

## Methods

### Ethical approval

All experimental procedures were previously approved by the Institutional Committee for Care and Use of Animals (CICUA) at the University of Chile (certificate 19266-VET-UCH).

### Experimental design

AT-MSCs and SCs were isolated from bovine fetuses (n=9) and adult bull testis (n=3) isolated from a local abattoir. Each pool represented a biological replicate and analyses, and experiments were performed thrice. Pooling tissue samples was performed in an effort to reduce individual biological variation. AT-MSCs and SCs were characterized accordingly to the gene expression of specific markers for MSC (+CD73, +CD90, and +CD105) and SC (+WT1 and +AR) using quantitative PCR (QPCR). The experiment involved incubating AT-MSCs in medium composed of 50% SCCM and 50% MSC medium (DMEM supplemented with 10% fetal bovine serum, FBS, 100 IU/mL penicillin, 100 μg/mL streptomycin and 2.5 μg/mL amphotericin B). The negative control consisted of AT-MSCs incubated in MSC medium. AT-MSCs were incubated in three replicates for 21 days at 38 °C with 5% CO_2_ under a humidified environment, with medium changes every 2 days. Cell culture samples were collected on days 0, 7, 14 and 21 of culture for determination of pluripotent (OCT4), GC (DAZL and PIWIL2) and meiotic (DMC1) marker gene expression using quantitative PCR (Q-PCR). Testicular extract was used as positive control for Q-PCR analyses. Samples were also collected on day 21 for quantification of pluripotent (OCT4) and GC (DAZL and PIWIL2) marker protein expression by flow cytometry analysis.

### Isolation and culture of bovine Sertoli cells (SCs)

Testes were decapsulated and testicular parenchyma was sliced and washed five times with HBSS. The tissue was allowed to sediment by gravity at room temperature for 15 min. The supernatant was removed, and the same procedure was repeated twice. After the final wash, HBSS was added in a 3:1 ratio and agitated at 300 x g at 37 °C for 20 min. The supernatant was removed and 10 mL of 2.5% trypsin, 10 mL of 1% Collagenase I, 2 mL of DNase I (10 mg/ml HBSS) (Sigma-Aldrich, Saint Louis, MO, USA) and 80 mL of HBSS were added. The tissue was agitated at 300 x g at 37 °C for 15 min. The enzymatic reaction was neutralized with DMEM-F12 medium (DMEM-F12 supplemented with 10% FBS, 100 IU/mL penicillin, 100 μg/mL streptomycin and 2.5 μg/mL amphotericin B). The cell suspension was centrifuged at 200 x g for 5 min. The supernatant was discarded and DMEM-F12 medium was added in a ratio of 10:1. The cell suspension was allowed to sediment by gravity for 30 min. The supernatant was then removed, and the sediment was resuspended and pipetted repeatedly in PBS (pH:7.4) supplemented with 1 M glycine and 2 mM EDTA for 10 min. The cellular content was allowed to sediment by gravity for 15 min. This procedure was repeated twice. The supernatant was removed and the enriched SCs sediment was centrifuged at 200 x g for 5 min and plated with DMEM-F12 medium at 38.5 °C under a humidified atmosphere with 5% CO_2_. After 48 hours, non-adherent cells were removed by changing the culture medium. Upon reaching 80-90% confluence, cells were detached using 0.5% trypsin in 0.2% EDTA and subjected to culture expansion. SCs were characterized by morphological features using a phase-contrast microscope and analyzing the presence of specific SC markers Wilms Tumor 1 (WT1) and Androgen Receptor (AR) through Q-PCR (Gaur et al., 2018).

### Isolation and culture of bovine fetal AT-MSCs

AT-MSCs were harvested following a previously reported protocol that ensured establishment of MSC cultures which expressed specific mesenchymal markers, confirming their identity (Segunda et al., 2019). Fetal AT was collected from the fetal omentum, washed with phosphate-buffered saline (PBS) (pH 7.4, Gibco, Grand Islands, NY, USA), minced and incubated in 0.5% collagenase I (1 mL/g of AT) for 45 min at 37 °C. The enzymatic reaction was neutralized with MSC medium. The disaggregated AT was filtered using 40 µm-pores filters and centrifuged at 1800 x g for 5 min. The cell pellet was resuspended in MSC medium and incubated at 38.5 °C in a humidified atmosphere with 5% CO_2_. After 48 hours, non-adherent cells were removed by changing the culture medium. Upon reaching 80-90% confluence, cells were detached using 0.5% trypsin in 0.2% EDTA and subjected to culture expansion.

### Preparation of Sertoli cell conditioned medium (SCCM)

Confluent SCs (5 x 10^4^ cells/cm^2^) were washed with PBS and cultured in serum-free DMEM-F12. After 72 hours of incubation, the medium was collected, centrifuged at 3000 x g for 10 min, filtered using a 0.22-um filter and stored at -80 °C until use (Dissanayake et al., 2018).

### *In vitro* germ cell differentiation induction protocol

As previously described, AT-MSCs were incubated in a medium composed of 50% SCCM (AT-MSC/SCCM) and 50% MSC medium in 6-well plates (Corning, NY, USA). Negative control consisted of AT-MSCs incubated in MSC medium. AT-MSCs were incubated for 21 days at 38 °C with 5% CO_2_ under a humidified environment, with medium changes every 2 days. Samples were collected on days 0, 7, 14 and 21 of treatment, fixed in lysis buffer supplemented with 2% β-Mercaptoethanol for Q-PCR or in 4% paraformaldehyde for flow cytometry (Cortez et al., 2018; Segunda et al., 2019).

### Gene expression analysis by quantitative PCR (Q-PCR)

Samples were analyzed for expression of endogenous genes β-ACTIN and GAPDH, MSC genes CD73, CD90 and CD105, pluripotency genes OCT4 and NANOG, GC genes FRAGILIS, STELLA, VASA, DAZL and STRA8, male GC genes PIWIL2 and SRY, and meiotic progression genes SCP3, REC8 and DMC1 by Q-PCR (Table 1). Total RNA was extracted using GeneJet RNA Purification kit (Thermo Fisher Scientific, CA, USA) following the manufacturer's instructions. The concentration of total RNA was quantified using Qubit 3.0 RNA assay kit (Fluorometer, CA, USA) and genomic DNA was removed using DNase I (Thermo Fisher Scientific, CA, USA). Complementary DNA (cDNA) was synthesized and amplified using Affinity Script Q-PCR cDNA Synthesis kit (Agilent Technologies, CA, USA), using a Step One thermocycler (Applied Biosystems, CA, USA). Thermal conditions were 25 °C for 5 min, 42 °C for 15 min and 95 °C for 5 min. Samples were subjected to RT-PCR using a Brilliant II SYBR Green QPCR Master Mix kit (Agilent Technologies, CA, USA) using an Eco Real-Time PCR System thermocycler (Illumina, CA, USA). Each PCR reaction tube contained 1 µL forward primer, 1 µL reverse primer, 5 µL Master Mix II SYBR Green, 5 ng cDNA and 2 µL nuclease-free water. Thermal cycling conditions were 95 °C for 10 min, followed by 40 repetitive cycles at 95 °C for 30 s and 60 °C for 1 min. Relative gene expression analysis was quantified using the comparative ∆∆Ct method (Ct: Threshold Value) (Vandesompele et al., 2002).

**Table 1:** Nucleotide sequence primers used for Q-PCR analysis.

Gene	Primer	Nucleotide sequence (5’-3’)	Accession number
**Endogenous**			
β-ACTINA	Forward	CGCACCACTGGCATTGTCAT	NM_173979.3
	Reverse	TCCAAGGCGACGTAGCAGAG	
GADPH			NM_001034034.2
	Forward	CCTTCATTGACCTTCACTACATGGTCTA	
	
	Reverse	TGGAAGATGGTGATGGCCTTTCCATTG	
**MSC**			
CD73	Forward	TGGTCCAGGCCTATGCTTTTG	BC_114093
	Reverse	GGGATGCTGCTGTTGAGAAGAA	
CD90	Forward	CAGAATACAGCTCCCGAACCAA	NM_001034765
	Reverse	CACGTGTAGATCCCCTCATCCTT	
CD105	Forward	CGGACAGTGACCGTGAAGTTG	NM_001076397
	Reverse	TGTTGTGGTTGGCCTCGATTA	
**Pluripotency**			
NANOG	Forward	TAAGCACAGGGGGCAAAAGT	NM_001025344.1
	Reverse	ATGGCTAAAAGGGGTGGAGG	
OCT4	Forward	GAAAGAGAAAGCGGACGA	NM_174580.3
	Reverse	GTGAAAGGAGACCCAGCA	
**GC**			
FRAGILIS	Forward	ACCTTCCTTGGTGGCTTTG	NM_001078142
	Reverse	TCACAGACAAGGGTGCTTTATT	
STELLA	Forward	TGCAAGTTGCCACTCAACTC	NM_00111110
	Reverse	TCTTACCCCTCTCCGCCTAT	
VASA	Forward	TGCTACTCCTGGAAGACTGA	NM_001007819.1
	Reverse	CGGTCTGCTGAACATCTCTA	
DAZL	Forward	TCCAAGTTCACCAGTTCAGG	NM_001081725.1
	Reverse	CGTCTGTATGCTTCTGTCCAC	
STRA8	Forward	TGTGCCCAGGTGTTCATCTC	XM_015463130
	Reverse	GGGGACTGTCACCTCATTGG	
**Male GC**			
PIWIL2	Forward	TCGTATTGATGATGTGGATTGG	XM_617223.3
	Reverse	GGGAGCAGCAGGATTTCAC	
SRY	Forward	AATAAGCACAAGAAAGTCCAGG	XM_015465855.1
	Reverse	CAAAAGGAGCATCACAGCAG	
**Meiotic**			
SCP3	Forward	GCTGGAAAGATTTGGAGCTG	BC_102433
	Reverse	ATCCCACTGCTGGAACAAAG	
DMC1	Forward	TCTCTCATACCCTCTGTGTG	NM_001191338.1
	Reverse	TTGTCCAGGACTGCATCATG	
REC8	Forward	AGCCTGCTTCTTCCTAACCA	XM_027553210.1
	Reverse	ACTTTCTCTGGGGTCACAGC	
**SC genes**			
WT1	Forward	CGTGCGTACCATGTAGGGAA	XM_015474834.2
	Reverse	CTCGTGCTTGAAGGAGTGGT	
AR	Forward	CAGATGGCAGTCATTCAG	XM_001244127
	Reverse	CTTGGTGAGCTGGTAGAAG	

### Flow cytometry

Determination of cell population positive for OCT4, DAZL and PIWIL2 was estimated using flow cytometry in AT-MSCs/SCCM and control AT-MSCs. Samples were centrifuged at 1800 x g for 5 min, resuspended in a solution of 1% Triton in PBS for 15 min at room temperature (RT), centrifuged at 1800 x g for 5 min and resuspended in 0.1% Tween in PBS. Antigens were blocked using a solution of 3% bovine serum albumin (BSA) in PBS with 1.5 mg/mL glycine for 1 h at RT. Cells were incubated in primaries mouse monoclonal anti-OCT4 antibody (Cat.#sc-5279; Santa Cruz Biotechnology, CA, USA) or rabbit polyclonal anti-DAZL (Cat.#ab34139; Abcam) or anti-PIWIL2 antibodies (Cat.#ab85084; Abcam) diluted (1:50) in 3% BSA in PBS overnight at 4 °C. Cells were washed thrice in 0.1% Tween in PBS, centrifuged at 1800 x g for 5 min and incubated in a solution of secondary goat anti-rabbit IgG conjugated with FITC (Cat#ab97050; Abcam, USA) or goat anti-mouse IgG conjugated with Alexa Fluor 488 (Cat#A21202; Thermo Fisher Scientific) diluted (1:1000) in 3% BSA in PBS for 2 h at 37 °C. Cells were resuspended in PBS and analyzed using a FACSCalibur Flow Cytometer (Becton Dickinson). Negative procedural control corresponded to cells incubated only with secondary antibody. Percentage of cells positive for secondary antibody was subtracted from the percentage of cells positive with primary and secondary antibodies.

### Statistical analysis

The statistical model considered relative gene expression and proportion of positive cells to markers as dependent variables, and the independent variables were days in culture, type of treatment (AT-MSCs/SCCM or control AT-MSCs) and the interaction between variables. A significance value of P<0.05 was used. Shapiro Wilks test was used to assess the normal distribution of data. Relative gene expression values were analyzed using Kruskal Wallis, and differences between means for culture days and treatments were determined using Dunn's post-test. The proportions of positive cells to markers were analyzed using a Student's t-test. All statistical analyses were performed using Info Stat software 2008 (Cordoba, Argentina).

## Results

### Isolation and characterization of bovine Sertoli cells

**Figure 1:**
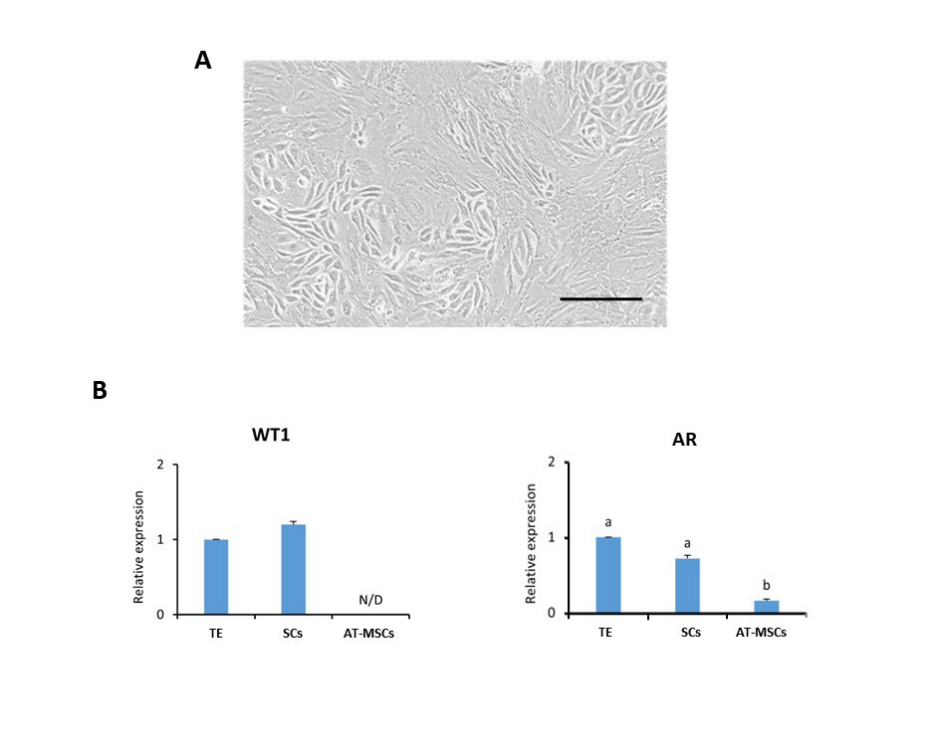
Phase contrast microscopy and relative gene expression of markers in bovine SC cultures. (A) SCs displayed fibroblast or polygonal morphology in culture; (B) WT1 gene expression was not different (P>0.05) between TE and SCs. However, WT1 was not detected in AT-MSCs. AR gene expression was higher (P<0.05) in TE and SCs compared to AT-MSCs. Different superscripts (a,b) indicate differences (P<0.05) between samples. Abbreviations: N/D (not detected); TE (testicular extract). Scale Bar (A): 100 µm.

Bovine SCs displayed adherence to plastic culture dishes with fibroblastic or cuboidal morphology (Figure 1A). The mRNA levels of SC markers WT1 and AR were quantified using Q-PCR analysis with the aim to evaluate homogeneity of SCs cultures. WT1 gene expression was detected in SCs and TE with no significant differences (P>0.05) between them; however, this marker was not detected in AT-MSCs (Figure 1B). AR gene expression was higher (P<0.05) in TE and SC compared to AT-MSCs (Figure 1C).

### Isolation and characterization of bovine fetal AT-MSCs

**Figure 2:**
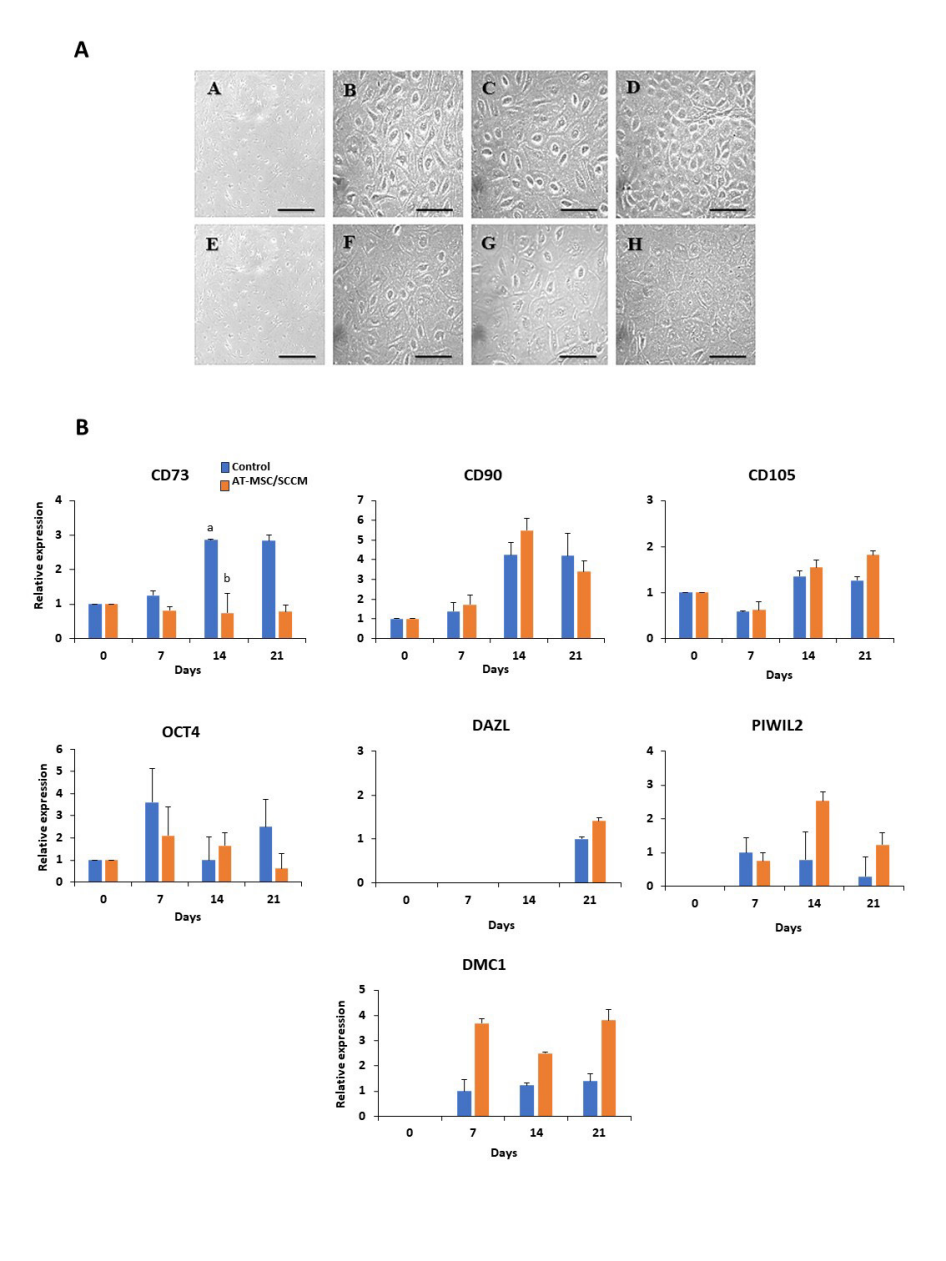
Phase contrast microphotographs and relative gene expression of specific MSC markers in AT-MSCs/SCCM and control during 21 days of culture. (A) AT-MSC/SCCM exhibit typical fibroblast-like morphology on day 0 (A) and mainly polygonal morphology from days 7, 14 and 21 of culture (B-D). Similarly, control AT-MSCs showed polygonal morphology from days 7, 14 and 21 of culture (F-H), with a slight less contrast compared to AT-MSCs/SCCM; (B) CD73 gene expression decreased (P<0.05) on day 14 in AT-MSCs/SCCM compared to control on the same day. Relative gene expression of CD90 and CD105 were not different (P>0.05) on days 7, 14 and 21 of culture compared to day 0 and controls. OCT4, DAZL and PIWIL2 gene expression were detected from days 0, 21 and 7, respectively; however, no differences (P>0.05) were detected between treatments or compared to day 0. DMC1 gene expression values were detected from day 7 to 21 with no significant differences between treatments. Scale Bar (A, E): 500 µm; (B-D, F-H): 100 µm. Superscripts (a,b) indicate a significant (P<0.05) difference between treatments or day 0.

Upon reaching 80-90% confluence, plastic-adherent bovine fetal AT-MSCs displayed typical fibroblast and polygonal-like morphology (Figure 2A). These cells were positive for mRNA expression of mesenchymal markers CD73, CD90 and CD105 and pluripotent gene OCT4 (Figure 2B); however, they did not express DAZL, PIWIL2 or DMC1.

### Morphological analysis of AT-MSC incubated in SCCM medium

During germinal differentiation, both cell types maintained a polygonal morphology throughout the culture period, with a slight increase in contrast observed in AT-MSC/SCCM compared to control AT-MSCs (Figure 2A).

### Gene expression of specific MSC markers in AT-MSCs cultured in SCCM for 21 days

CD73 relative gene expression was lower (P<0.05) in AT-MSCs/SCCM on day 14 of culture compared to their respective control (0.74-fold versus 2.86-fold); however, no differences were found (P>0.05) on days 7 and 21 (Figure 2B; Table 2). CD90 and CD105 relative gene expression was detected in AT-MSC/SCCM on days 0, 7, 14, and 21 of culture; however, no significant differences were found between AT-MSCs/SCCM and control (Figure 2B; Table 2).

**Table 2:** Expression profile of MSC, GC, male GC and meiotic progression markers in AT-MSC/SCCM analyzed by Q-PCR and flow cytometry.

**Marker**	**AT-MSC/SCCM**
CD73	↓ (Q-PCR)
CD90	* (Q-PCR)
CD105	* (Q-PCR)
OCT4	* (Q-PCR)
	* (FC)
NANOG	**x** (Q-PCR)
FRAGILIS	**x** (Q-PCR)
STELLA	**x** (Q-PCR)
VASA	**x** (Q-PCR)
DAZL	* (Q-PCR)
	* (FC)
STRA8	**x** (Q-PCR)
PIWIL2	* (Q-PCR)
	* (FC)
SRY	**x** (Q-PCR)
SCP3	**x** (Q-PCR)
REC8	**x** (Q-PCR)
DMC1	* (Q-PCR)

Abbreviations: ↓, decreased expression compared to control on day 14 of culture; *no significant differences (P>0.05) compared to control; x: not detected; Q-PCR: Quantitative PCR; FC: Flow Cytometry.

### Expression of pluripotency and specific GC markers in AT-MSC cultured in SCCM for 21 days

**Figure 3:**
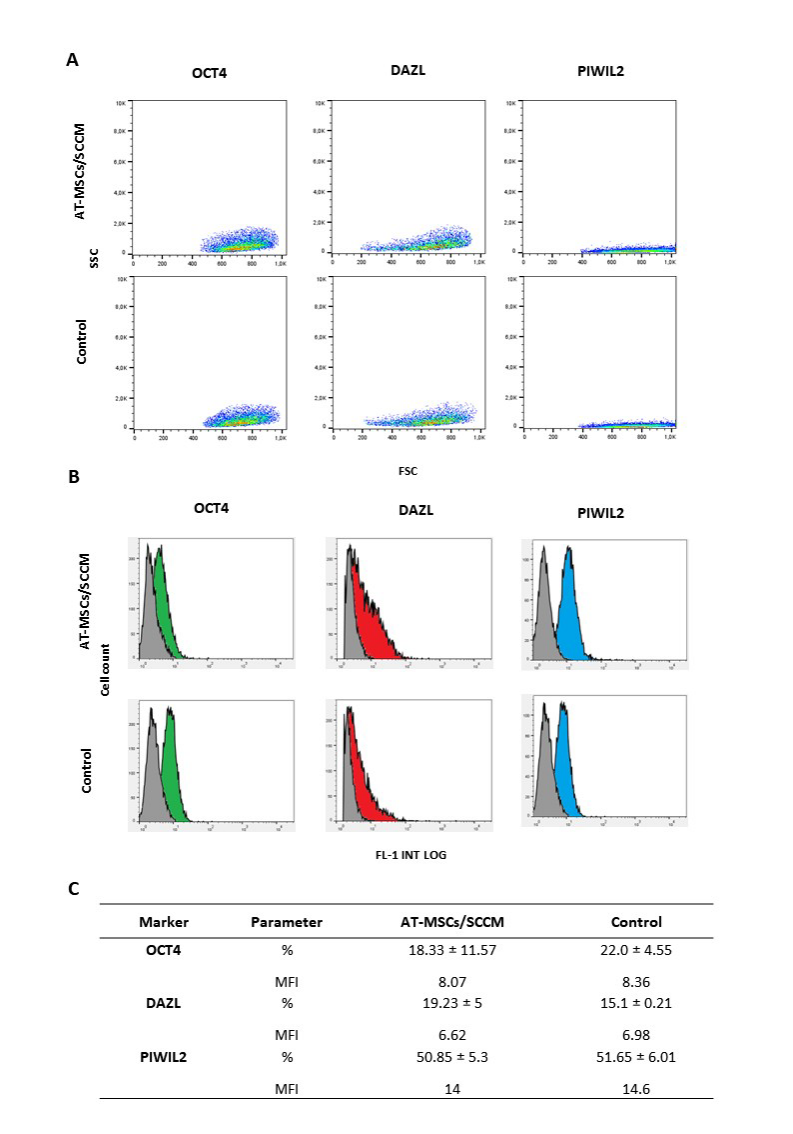
Expression of OCT4, DAZL and PIWIL2 markers in AT-MSC/SCCM and control during 21 days of culture. (A) Representative dot plots showing distribution of cells positive for OCT4, DAZL and PIWIL2 in AT-MSC/SCCM and control based on size (FSC) and complexity (SSC) at day 21 days of culture; (B) Representative histograms displaying cells positive for OCT4 (green area), DAZL (red area) and PIWIL2 (blue area) in AT-MSC/SCCM and control at day 21 of culture. Gray area represents cells incubated only with secondary antibody (C) OCT4, DAZL, PIWIL2-positive cells were detected in both AT-MSC/SCCM and control, with no differences (P>0.05) between them. Abbreviations: (MFI) Mean Fluorescence Intensity; (FL1-INT-LOG) signal intensity of protein expression on a logarithmic scale.

OCT4 gene expression was detected in AT-MSC/SCCM on days 0, 7, 14 and 21 of culture. However, no differences were found (P>0.05) between AT-MSC/SCCM and their respective controls (2.1, 1.64 and 0.62-fold versus 3.6-, 1- and 2.5-fold day 0) (Figure 3B; Table 2). The presence of OCT4-positive cells was detected in both AT-MSC/SCCM and control AT-MSC, with no differences (P>0.05) between the two culture conditions (%: 18.33 ± 11.57; MFI: 8.07 versus %: 22 ± 4.55; MFI: 8.36) (Figure 3A-C; Table 3). DAZL gene activation was detected in AT-MSCs/SCCM on day 21 of culture, but its expression was not different (P>0.05) compared to control AT-MSCs on the same day (1.4-fold versus 1-fold) (Figure 3B; Table 2). The presence of cells positive for DAZL was detected, with no differences (P>0.05) between AT-MSCs/SCCM and control AT-MSCs (%: 19.23 ± 5; MFI: 6.62 versus %: 15.1 ± 0.21; MFI: 6.98) (Figure 3A-C; Table 2). PIWIL2 gene expression was detected in AT-MSCs/SCCM on days 7, 14 and 21 of culture, but no differences were found (P>0.05) compared to control AT-MSCs on day 7 (0.75-, 2.53-, 1.23-fold versus 1-, 0.78-, 0.29-fold day 0) (Figure 2B; Table 2). The presence of cells positive for PIWIL2 was detected in both AT-MSCs/SCCM and control AT-MSCs, with no differences (P>0.05) between the two culture conditions (%: 50.85 ± 5.3; MFI: 14 versus %: 51.65 ± 6.01; MFI: 14.6) (Figure 3C; Table 2). DMC1 gene expression was activated in AT-MSC/SCCM and control AT-MSC on days 7, 14, and 21 of culture with no significant differences between treatments (4.1-, 3.1-, 4.1-fold versus 1.1-, 1.2-, 1.3-fold day 0) (Figure 3D; Table 2).

**Table 3:** Population of markers-positive cells and mean fluorescence intensity for OCT4, DAZL and PIWIL2 in AT-MSC/SCCM and control AT-MSC.

**Marker**	**Parameter**	**AT-MSC/SCCM**	**Control AT-MSC**
**OCT4**	%	18.33 ± 11.57	22.0 ± 4.55
	MFI	8.07	8.36
**DAZL**	%	19.23 ± 4.99	15.1 ± 0.21
	MFI	6.62	6.98
**PIWIL2**	%	50.85 ± 5.3	51.65 ± 6.01
	MFI	14.0	14.6

Abbreviations: MFI (Mean Fluorescence Intensity).

## Discussion

In this study, AT-MSCs were isolated by their adherence to plastic flasks during cell culture and were characterized by gene expression of MSC markers including CD73, CD90 and CD105. According to the International Society for Cellular Therapy (ISCT), these parameters are among the minimum criteria for definition of human MSCs; however, definition for bovine MSCs has not been declared (Dominici et al., 2006). Bovine fetal AT-MSCs were isolated using a protocol based on enzymatic digestion and plastic adherence. Using similar isolation protocols, previous reports have indicated that bovine AT-MSCs were able to express MSC markers and display trilineage differentiation potential including adipogenic, osteogenic and chondrogenic phenotypes (Lu et al., 2014; Oyarzo et al., 2021; Yaneselli et al., 2024). This report suggests that the isolation protocol used in our study allows the recovery of populations of AT-MSCs with the potential for trilineage differentiation. SCs were characterized by gene expression of WT1 and AR, where WT1 was specific in SCs and AR mRNA levels were higher compared to AT-MSCs and TE. These results suggest that the isolation protocols for AT-MSCs and SCs were efficient and resulted in homogenous cultures.

The use of SCCM to induce GC differentiation has been previously described (Ghaem et al., 2018; Dissanayake et al., 2018; Monfared et al., 2016). Despite this strategy lack direct contact between GCs and SCs, its effect is based on all the factors secreted by SCs, which mimic the molecular mechanisms that regulate spermatogenesis *in vivo*. Some of the factors present in the SCCM include BMP4, leukemia inhibitory factor (LIF), basic fibroblast growth factor (bFGF), SCF, growth differentiation factor-9 (GDF-9), glial cell line-derived neurotrophic factor (GDNF), among others, which are involved in maintenance of viability, proliferation and differentiation of the GCs (Huang et al., 2010; Monfared et al., 2016). Although previous studies have supplemented SCCM with retinoic acid (Ghaem et al., 2018; Dissanayake et al., 2018), our study was similar to a report where BM-MSCs were treated sole with SCCM (Monfared et al., 2016). Despite the use of sole SCCM induced expression of GC marker MVH, a more robust GC gene expression profile was obtained when SCCM was supplemented with retinoic acid (Ghaem et al., 2018; Dissanayake et al., 2018). This GC gene expression profile included down-regulation of expression of pluripotency markers OCT4 and NANOG and up-regulation of premeiotic STRA8, meiotic SCP3, and postmeiotic ACR and PRM1 genes (Ghaem et al., 2018). Furthermore, our analyses have reported that supplementation of DMEM with retinoic acid increase expression of DAZL and regulation of OCT4 and NANOG (Cortez et al., 2018). These results suggest that retinoic acid may play a crucial role in GC differentiation and that supplementation of SCCM with retinoic acid may be a useful strategy to improve GC differentiation of MSCs in cultures systems based on SCCM.

During GC differentiation, AT-MSC/SCCM and controls maintained a polygonal shape throughout the culture period, with a slight increase in contrast observed in AT-MSC/SCCM compared to control. This result was different from other assays that cultured MSCs with SCCM, which reported increasingly more rounded cells, a change associated with the presence of round spermatocytes and spermatids (Dissanayake et al., 2018). These changes could be associated with the effect of the secretome of SCCM, which corresponds to the set of molecules released from SCs into the extracellular environment (Műzes and Sipos, 2022). In this regard, SC secretome could contain different molecules that induce slight changes in AT-MSCs, changes that were not detected in control, including BMP4, whose receptors are expressed in spermatogonias, exerting a differentiating effect on them (Rossi and Dolci, 2013; Yang et al., 2016); C-KIT, which binds to its ligand SCF, produced by SCs, which stimulate the differentiation of spermatogonia into spermatocytes (Unni et al., 2009); TGFβ3, whose function is implicated in the differentiation of GCs (Lui et al., 2003; Skinner and Griswold, 2005); and RALDH1 enzyme, which metabolizes retinol into RA (Teletin et al., 2019), inducing the differentiation of spermatogonias by promoting STRA8 gene expression (Rossi and Dolci, 2013). It is likely that some of these molecules are responsible for affecting the contrast of MSC cultures, however, since the composition of SCCM is not fully defined, further research is needed to analyze its components and its effect on AT-MSC.

In this study, CD73 gene expression was decreased on day 14 of culture in AT-MSC/SCCM compared to control. CD73 is a surface marker for MSC with functions related to cell proliferation and adhesion (Hu et al., 2023). Although CD73 is considered a classic surface marker for defining MSC, treatments have been described to modulate expression of surface markers in MSC (Nakamura et al., 2003; Hung et al., 2004). In this study, the decrease in CD73 gene expression in AT-MSC/SCCM could be influenced by SCCM, whose molecules may potentially affect the expression of CD73. This decrease may have caused a disruption in its functions by day 14 of culture; however, as no significant differences were detected at day 21, this result does not necessarily indicate a that AT-MSCs may potentially loss their mesenchymal profile and progressed into GC differentiation. CD90 and CD105 gene expression was detected in both AT-MSC/SCCM and control AT-MSC during the 21 days of culture and no significant differences were found between the two culture conditions. CD90 high expression has been associated with undifferentiated MSC and some studies suggest its function in MSC self-renewal and differentiation (Moraes et al., 2016). CD105 is a component of the TGFβ receptor complex, with functions related to promoting chondrogenesis, stimulating angiogenesis and scar remodeling (Czapla et al., 2016; Wang et al., 2020b). The results of this study indicate that CD90 and CD105 gene expression were not affected by the culture time or exposure to SCCM, suggesting that AT-MSCs maintained their mesenchymal profile throughout the culture period.

OCT4 gene expression was detected from day 0 to day 21 of culture, with a proportion of positive cells on day 21 in both AT-MSC/SCCM and control. However, no significant differences were observed between both treatments. OCT4 is a transcription factor involved in establishing and maintaining pluripotency in embryonic stem cells (ESC) (Do et al., 2014) and it is not considered a marker of pluripotency in MSC, which are multipotent cells (Williams and Hare, 2011). However, its presence could indicate a degree of cellular pluripotency. Nevertheless, concordance between QPCR and flow cytometry results suggested that treatment with SCCM did not induce a change in the expression of OCT4 marker.

An activation of DAZL was observed on day 21, and a proportion of positive cells was detected on day 21 in both AT-MSC/SCCM and control, with no significant differences between two treatments. DAZL is one of the most important GC markers and it can be detected throughout the life of GCs, being necessary for germinal differentiation, growth and maturation (Yen, 2004; Park et al., 2013; Ghasemzadeh-Hasankolaei et al., 2014a). This result suggest that the microenvironment induced by SCCM and MSC medium may have an inductive effect on DAZL transcription, with the common factor in both cultures being FBS, which could contain factors that activate DAZL in AT-MSC. Some of these factors could be the hormone progesterone, which increases DAZL expression in treated cells (Moghaddam et al., 2021), or testosterone, a hormone that exerts its effects on spermatogenesis through the expression of proliferation and differentiation factors (Parekh et al., 2019; Lee et al., 2022).

Activation of PIWIL2 was observed on day 7 of culture, which persisted until day 21. Additionally, a proportion of PIWIL2-positive cells was detected on day 21 of culture in both AT-MSCs/SCCM and control, with no significant differences between them. PIWIL2 is exclusively expressed in spermatogonia and plays a role in GCs self-renewal and differentiation of spermatogonia into spermatocytes (Drusenheimer et al., 2007; Ghasemzadeh-Hasankolaei et al., 2014b). The activation of PIWIL2 could also be influenced by the hormonal effect present in FBS, like DAZL, as both markers are specific to GCs, suggesting a similar inductive effect.

Activation of DMC1 was observed from day 7 of culture in both AT-MSC/SCCM and control, with no significant differences between groups. DMC1 encodes a protein necessary for cross-over recombination of chromosomes during the meiotic division (Nimonkar et al., 2012). Since this marker is specific to GCs, its activation in both types of cultures is also related to what happened with DAZL and PIWIL2, meaning that their inductive factor may be associated to FBS effect.

In this study, GC differentiation process of AT-MSCs cultured in SCCM was analyzed using Q-PCR and flow cytometry. Expression of CD73, CD90, CD105, OCT4, DAZL, PIWIL2 and DMC1 in both treated and control cells, suggests that SCCM was not capable of inducing the differentiation of AT-MSC into male GCs. The expression levels of markers in AT-MSC/SCCM and control did not show significant differences, which could be associated to the action of FBS present in both types of cultures, acting as a GC differentiation factor. FBS is a supplement used in cell culture media, as it contains abundant micro and macronutrients, growth factors and hormones that promote cell adhesion, growth and proliferation (Subbiahanadar et al., 2021). However, the composition of FBS has not been fully determined, causing heterogeneity between assays (Guiotto et al., 2020). Many factors present in FBS including bFGF, GDNF y TGFβ1 (Lee et al., 2022) have also been involved in GC differentiation. Moreover, hormones present in FBS including estradiol, progesterone and testosterone may induce the transcription of specific male GC markers at both gene and protein levels, which may have masked the true effect of SCCM on AT-MSC.

## Conclusion

The expression profiles of GC markers and their similarity between both culture conditions indicate that AT-MSC did not initiate the differentiation process into GCs or meiotic progression under the influence of SCCM.

## Data Availability

The datasets used and/or analyzed during the current study are available from the corresponding author on reasonable request.
